# Undercarboxylated Osteocalcin and Its Associations With Bone Mineral Density, Bone Turnover Markers, and Prevalence of Osteopenia and Osteoporosis in Chinese Population: A Cross-Sectional Study

**DOI:** 10.3389/fendo.2022.843912

**Published:** 2022-07-08

**Authors:** Yang Xu, Li Shen, Lianyong Liu, Zhenlin Zhang, Weiwei Hu

**Affiliations:** ^1^ Shanghai Clinical Research Center of Bone Disease, Department of Osteoporosis and Bone Diseases, Shanghai Jiao Tong University Affiliated Sixth People’s Hospital, Shanghai, China; ^2^ Clinical Research Center, Shanghai Jiao Tong University Affiliated Sixth People’s Hospital, Shanghai, China; ^3^ Department of Endocrinology, Punan Hospital of Pudong New District, Shanghai, China

**Keywords:** undercarboxylated osteocalcin, bone mineral density, bone turnover markers, osteopenia, osteoporosis

## Abstract

**Objective:**

Undercarboxylated osteocalcin (ucOC) is one form of osteocalcin lacking full carboxylation, which plays an important role in bone homeostasis, glucose homeostasis, and energy metabolism. Our aim is to obtain the profile of serum ucOC level according to gender and age and explore its associations with bone mineral density (BMD), bone turnover markers (BTMs), and prevalence of osteopenia and osteoporosis in the Chinese population.

**Methods:**

This is a cross-sectional study with 900 subjects, composed of 431 men and 469 women. Clinical information was collected, and BMD values of the lumbar spine (L1–4), left femoral neck, and total hip were scanned. Biochemical markers including hepatic and renal function, serum calcium, serum phosphorus, procollagen type 1 N-propeptide (P1NP) β-CrossLaps of type I collagen-containing cross-linked C-telopeptide (β-CTX) intact parathyroid hormone (PTH), 25-hydroxyvitamin D (25OHD), and ucOC were measured.

**Results:**

We found that the median ucOC level was higher in men than women [men, 2.6 ng/ml; women, 1.6 ng/ml; *p* < 0.001]. The profile according to age showed that ucOC levels were the lowest at the age of 40–49 years in both men [2.55 ng/ml (95% CI = 1.96–3.13 ng/ml)] and women [1.57 ng/ml (95% CI = 1.12–2.03 ng/ml)]; in patients younger than 49 years, they decreased with age; then over 50 years, they quickly increased. Furthermore, we found that a higher ucOC level was correlated with lower BMD values at the lumbar spine (men, r = −0.128, *p* = 0.013; women, r = −0.321, *p* < 0.001), femoral neck (men, r = −0.095, *p* = 0.062; women, r = −0.260, *p* < 0.001), and total hip (men, r = −0.123, *p* = 0.015; women, r = −0.209, *p* < 0.001) and higher P1NP (men, r = 0.307, *p* < 0.001; women, r = 0.239, *p* < 0.001) and β-CTX (men, r = 0.169, *p* = 0.001; women, r = 0.354, *p* < 0.001) levels in both men and women. Furthermore, we also showed that a 1 − SD increase in ucOC was associated with an odds ratio (OR) of 1.63 and 1.70 for having osteopenia or osteoporosis in men and women, respectively (men, 95% CI = 1.25–2.13, *p* = 0.004; women, 95% CI = 1.19–2.42, *p* = 0.004).

**Conclusions:**

We first revealed the profile of serum ucOC levels according to gender and age in the Chinese population and demonstrated the associations of ucOC with BMD and BTMs and the risk of prevalent osteopenia or osteoporosis. Our findings provide a clue to elucidate the function of ucOC in bone metabolism.

## Introduction

Osteopenia and osteoporosis are common diseases worldwide, which are associated with increased fracture risk, especially in the aging population (1–4). Early detection and intervention for individuals with osteopenia or osteoporosis may effectively reduce the socioeconomic burden caused by fractures (1, 4, 5). Bone mineral density (BMD) is used as an important indicator for the diagnosis of osteopenia and osteoporosis, while bone turnover markers (BTMs) are a useful adjunct for therapeutic monitoring (6, 7). Osteocalcin (OC) is one of the traditional BTMs, which reflects skeletal status and bone remodeling (8, 9). Specially synthesized and secreted by osteoblasts, OC is an abundant non-collagenous protein (10). During the process of post-translational modification, 17, 21, and 24 glutamic acid residues of OC undergo carboxylation mediated by vitamin-K-dependent γ-glutamyl carboxylase (GGCX), which leads to a conformational change with stable α-helical portion and greater affinity for calcium and hydroxyapatite (11, 12). According to the degree of carboxylation, OC presents in two forms: fully carboxylated OC (cOC) and undercarboxylated OC (ucOC) lacking carboxylation at one or more sites (13, 14). Of the total amount of OC in circulation, 40%–60% is ucOC.

Recently, Smith et al. (15) revealed the “U shaped” relationship between ucOC and age in Australian adult men; however, there were no age-related studies on ucOC levels in Chinese men or women. In addition, recent advances have indicated that ucOC not only is a nutritional biomarker sensitive to vitamin K status but also functions as a hormonally active isoform in the regulation of glucose homeostasis and energy metabolism (16). Recent studies have shown that ucOC is a useful marker to identify the increased metabolic syndrome risk and a potential therapeutic target for cardiovascular diseases (17–19). Regarding the field of bone homeostasis, ucOC is released into circulation due to its reduced affinity to the bone matrix. Vergnaud et al. (20) found that serum ucOC predicted the risk of hip fracture independent of femoral neck BMD in older women. Natsumi et al. (21) revealed that vitamin K deficiency and high serum ucOC were correlated with poor bone status in women. Furthermore, some studies have demonstrated that higher serum ucOC levels were associated with lower BMD in women (22–24). Yamauchi et al. (25) have found that ucOC levels were positively correlated with urinary type I collagen cross-linked-N-telopeptide (uNTX), but not BMD in postmenopausal women. Most of the research is performed in women, and there have been few reports on correlations of serum ucOC with BMD and BTMs in men. Although a higher ucOC level was found in individuals with osteoporosis, it is needed to further investigate the relationship between the ucOC level and prevalent osteopenia and osteoporosis (26).

Therefore, in this cross-sectional study, we determined how ucOC levels change with gender and age in the Chinese population and defined the associations of ucOC with BMD, BTMs, and the prevalence of osteopenia and osteoporosis in both Chinese men and women.

## Materials and Methods

### Study Population

This study was approved by the Ethics Committee of the Shanghai Jiao Tong University Affiliated Sixth People’s Hospital. From November 2017 to March 2020, 934 individuals were recruited from the outpatient clinic, and written informed consent was obtained from all the participants. All participants were evaluated by a questionnaire about their medical history and fracture history, physical examination, and routine serum measurements including hepatic and renal function and serum calcium and phosphorus. Among the 934 participants, subjects with following conditions were excluded: 1) could not complete the questionnaire, physical examination, or routine serum measurements independently; 2) diseases affecting bone metabolism, including osteogenesis imperfecta, Paget’s disease of bone, rheumatoid arthritis, primary hyperparathyroidism, diabetes mellitus, or malignant tumors; 3) medication use affecting bone metabolism, including treatment with synthetic steroid hormones, epinephrine, denosumab, bisphosphonate, teriparatide, estrogen, or other anti-osteoporosis drugs in the past 1 year; 4) medication use including antacids containing aluminum, warfarin, and thrombolytic drugs; 5) serious primary diseases affecting the cardiovascular, pulmonary, hematopoietic, gastrointestinal, renal, or nervous systems or mental state; 6) abnormal biochemical measurements, including alkaline phosphatase (ALP), alanine aminotransferase (ALT), aspartate aminotransferase (AST), creatinine (Cr), uric acid (UA), blood urea nitrogen (BUN), serum calcium, and phosphorus. Eight subjects were excluded because they had taken bisphosphonate or teriparatide to treat osteoporosis, and another 26 subjects were excluded because of abnormal hepatic or renal function or serum calcium or phosphorus level. Finally, a total of 900 participants (aged 17–89 years; men, 431; women, 469) were enrolled in the study.

### Biochemical Measurements

Blood samples were collected from fasting participants in the morning from 7:00 a.m. to 10:00 a.m., and serum aliquots were stored at −80°C until being assayed. Routine hepatic and renal functions, including ALP, ALT, AST, Cr, UA, and BUN, and serum calcium and phosphorus were measured using a cobas c 701 automatic biochemistry analyzer (Roche Diagnostic GmbH, Basel, Switzerland). All the bone metabolism markers were measured by electrochemiluminescence immunoassay with the following kits (Roche Diagnostic GmbH): total P1NP kit for procollagen type 1 N-propeptide (P1NP), β-CrossLaps kit for b-CrossLaps of type I collagen-containing cross-linked C-telopeptide (β-CTX), intact PTH kit for parathyroid hormone (PTH), and vitamin D total kit for 25-hydroxyvitamin D (25OHD). Serum ucOC was measured by ELISA using monoclonal mouse anti-Glu-OC as the capture antibody, peroxidase-labeled monoclonal mouse anti-OC as the detection antibody, and tetramethylbenzidine for chromogenic reaction (Glu-OC kit, Takara Bio Inc., Otsu, Japan). The intra- and interassay coefficients of variation (CVs) were 5.2% and 8.3%, respectively.

### Other Measurements

Height and body weight were measured on an ultrasonic body scale. Body mass index (BMI) was calculated by dividing weight by squared height. The BMD at the lumbar spine (L1–4), left femoral neck, and total hip was scanned using dual-energy X-ray absorptiometry (DAX, Lunar Corp., Cambridge, MA, USA). Then, according to the WHO criteria for the classification of osteopenia and osteoporosis based on T or Z values of BMD and osteoporotic fractures, all the subjects were divided into two groups (27). The group of osteopenia or osteoporosis was assessed as follows: 1) T values of BMD at the lumbar spine, femoral neck, and total hip in the elderly men over 50 years or postmenopausal women < −1.0; 2) Z values of BMD at the lumbar spine, femoral neck, and total hip in men under 50 years or premenopausal women ≤ −2.0; and 3) with osteoporotic fractures in hip or spine and age over 50 years. Then, the remaining patients were considered as the group of normal BMD.

### Statistical Analysis

The Kolmogorov–Smirnov test was used to measure the normality of the data. The skewed distributed variables were expressed as median (interquartile range) and categorical variables as frequency (%). The Mann–Whitney U test was used to compare the differences between groups with non-normally distributed data, and the χ^2^ test or Fisher’s exact test was used for categorical variables. Partial correlation analysis was used to examine the associations of ucOC level with BMD and BTMs after adjusting for age, BMI, Cr, and 25OHD. The logistic regression model was used to estimate the odds ratio (OR) with 95% CIs for osteopenia or osteoporosis.

All statistical analyses were performed in the SPSS software (version 26.0, SPSS Inc. of IBM, USA) and R statistical software (version 4.0.2, R Foundation for Statistical Computing). A two-tailed statistical measure was used, with a *p* < 0.05 considered statistically significant.

## Results

### General Characteristics of the Study Population

The basic characteristics of the 900 participants are shown in **Table 1**. All the participants (men, 431; women, 469) had a median age of 48.0 years (interquartile range, 32.0–65.0 years), a median BMI of 22.9 kg/m^2^ (interquartile range, 20.8–25.2 kg/m^2^), and a median serum ucOC level of 2.0 ng/ml (interquartile range, 0.9–3.9 ng/ml).

**Table 1:** Basic characteristics of the 900 participants.

Characteristics	Total sample(n = 900)	Men(n = 431)	Women(n = 469)	*p*	*p* ^a^
Age (years)	48.0 (32.0–65.0)	52.0 (32.0–69.0)	46.0 (33.0–62.0)	**0.006**	**–**
BMI (kg/m^2^)	22.9 (20.8–25.2)	23.7 (21.6–26.0)	22.2 (20.3–24.4)	**<0.001**	**–**
Cr (μmol/L)	72.0 (63.0–84.0)	83.0 (72.0–93.0)	65.0 (59.0–73.0)	**<0.001**	–
25OHD (μg/L)	21.4 (15.0–29.0)	21.0 (16.0–29.0)	22.0 (15.0–29.5)	0.706	–
Lumbar spine BMD (g/cm^2^)	1.01 (0.90–1.15)	1.01 (0.91–1.15)	1.00 (0.88–1.15)	0.533	0.542
Femoral neck BMD (g/cm^2^)	0.82 (0.73–0.91)	0.84 (0.75–0.92)	0.81 (0.72–0.90)	**0.003**	**0.016**
Total hip BMD (g/cm^2^)	0.90 (0.80–0.99)	0.92 (0.82–1.01)	0.89 (0.78–0.97)	**<0.001**	**0.010**
ALP (U/L)	67.0 (54.0–83.0)	68.0 (55.0–84.0)	66.0 (52.8–82.3)	0.067	0.386
ALT (U/L)	13.0 (9.0–19.0)	13.0 (9.0–19.8)	13.0 (10.0–19.0)	0.632	0.371
AST (U/L)	19.0 (16.0–24.0)	20.0 (16.0–25.0)	19.0 (15.0–23.5)	0.072	0.178
UA (μmol/L)	312.0 (262.0–372.0)	340.0 (279.0–405.0)	294.0 (246.0–347.0)	**<0.001**	0.200
BUN (mmol/L)	5.0 (4.2–6.0)	5.2 (4.4–6.2)	4.9 (4.0–5.8)	**<0.001**	**<0.001**
Calcium (mmol/L)	2.42 (2.26–2.59)	2.35 (2.18–2.52)	2.47 (2.34–2.64)	**<0.001**	**<0.001**
Phosphorus (mmol/L)	1.20 (1.04–1.35)	1.08 (0.96–1.23)	1.30 (1.17–1.42)	**<0.001**	**<0.001**
P1NP (ng/ml)	46.3 (34.2–62.6)	47.0 (34.7–65.3)	45.9 (33.6–60.6)	0.122	0.892
β-CTX (ng/ml)	0.39 (0.27–0.56)	0.41 (0.27–0.58)	0.38 (0.27–0.55)	0.311	0.055
PTH (ng/L)	39.2 (28.9–51.0)	36.9 (25.9–49.0)	41.2 (31.2–52.8)	**<0.001**	**<0.001**
ucOC (ng/ml)	2.0 (0.9–3.9)	2.6 (1.3–4.7)	1.6 (0.7–3.1)	**<0.001**	**<0.001**

BMI, body mass index; BMD, bone mineral density; ALP, alkaline phosphatase; ALT, alanine aminotransferase; AST, aspartate aminotransferase; UA, uric acid; BUN, blood urea nitrogen; Cr, creatinine; P1NP, procollagen type 1 N-propeptide; β-CTX, β-CrossLaps of type I collagen-containing cross-linked C-telopeptide; 25OHD, 25-hydroxyvitamin D; PTH, parathyroid hormone; ucOC, undercarboxylated osteocalcin.

a Adjusted for age, BMI, Cr, and 25OHD. Significant values (p < 0.05) are presented in bold.

### The Profile of Serum Undercarboxylated Osteocalcin Level According to Gender and Age

From the 900 participants, men had a higher serum ucOC level than women after adjusting for age, BMI, Cr, and 25OHD [men, 2.6 ng/ml (interquartile range, 1.3–4.7 ng/ml); women, 1.6 ng/ml (interquartile range, 0.7–3.1 ng/ml); *p* < 0.001] (**Table 1**). In the line chart with the profile of serum ucOC according to age, after adjusting for BMI, Cr, and 25OHD, ucOC levels were the lowest in the age group of 40–49 years in both men [2.55 ng/ml (95% CI = 1.96–3.13 ng/ml)] and women [1.57 ng/ml (95% CI = 1.12–2.03 ng/ml)] (**Supplementary Figure S1**). In men, the ucOC level decreased with increasing age until 49 years and then quickly increased over the age of 50 years (**Table 2**). A similar tendency was also observed in women (**Table 2**).

**Table 2:** Means and 95% CI for the age-specific measurements of ucOC in men and women.

age	Men (n = 431)		Women (n = 469)	
	Number	ucOC	Number	ucOC
≤29	80	3.61 (2.87, 4.35)	86	3.23 (2.53, 3.93)
30–39	69	3.01 (2.42, 3.60)	95	1.89 (1.41, 2.37)
40–49	53	2.55 (1.96, 3.13)	79	1.57 (1.12, 2.03)
50–59	54	3.72 (2.96, 4.47)	72	2.15 (1.56, 2.74)
60–69	72	3.07 (2.42, 3.71)	78	2.46 (1.86, 3.06)
≥70	103	3.66 (3.21, 4.11)	59	1.73 (1.12, 2.33)

Adjusted for BMI, Cr, and 25OHD.

ucOC, undercarboxylated osteocalcin; BMI, body mass index; Cr, creatinine; 25OHD, 25-hydroxyvitamin D.

### The Associations of Serum Undercarboxylated Osteocalcin With Bone Mineral Density and Bone Turnover Markers

After age, BMI, Cr, and 25OHD were adjusted, partial correlation analysis revealed that a higher ucOC level was correlated with lower BMD at the lumbar spine (men, r = −0.128, *p* = 0.013; women, r = −0.321, *p* < 0.001), femoral neck (men, r = −0.095, *p* = 0.062; women, r = −0.260, *p* < 0.001), and total hip (men, r = −0.123, *p* = 0.015; women, r = −0.209, *p* < 0.001) in both men and women (**Table 3**). Additionally, a higher ucOC level was found to be correlated with higher P1NP levels in both men and women (men, r = 0.307, *p* < 0.001; women, r = 0.239, *p* < 0.001) and β-CTX (men, r = 0.169, *p* < 0.001; women, r = 0.354, *p* < 0.001) (**Table 3**). Since vitamin D is one of the main drivers of bone mineralization, we further evaluated the correlation of ucOC with BMD and BTMs in the vitamin D-sufficient subjects (25OHD ≥ 20 μg/L; men, 239; women, 273), and it revealed similar results as above (**Supplementary Table S1**).

**Table 3:** Correlations between serum ucOC level with BMD and BTMs.

Variables	Men (n = 431)		Women (n = 469)	
	r	p	r	p
Lumbar spine BMD (g/cm^2^)	−0.128	**0.013**	−0.321	**<0.001**
Femoral neck BMD (g/cm^2^)	−0.095	0.062	−0.260	**<0.001**
Total hip BMD (g/cm^2^)	−0.123	**0.015**	−0.209	**<0.001**
P1NP (ng/ml)	0.307	**<0.001**	0.239	**<0.001**
β-CTX (ng/ml)	0.169	**0.001**	0.354	**<0.001**

Adjusted for age, BMI, Cr, and 25OHD, and significant values (p < 0.05) are presented in bold.

BMD, bone mineral density; P1NP, procollagen type 1 N-propeptide; β-CrossLaps of type I collagen-containing cross-linked C-telopeptide.

### The Associations of Serum Undercarboxylated Osteocalcin With Prevalence of Osteopenia or Osteoporosis

According to WHO criteria for the classification of osteopenia and osteoporosis, we classified all participants with accessible BMD values and osteoporotic fracture information into two subgroups: one with osteopenia or osteoporosis (N = 386; men, 190; women, 196) and another subgroup with normal BMD (N = 493; men, 221; women, 272). Twenty-one subjects without exact BMD values were excluded. As shown in **Table 4**, the logistic regression model with age, BMI, Cr, and 25OHD adjusted revealed that a 1 − SD increase in ucOC was associated with an OR of 1.63 and 1.70 for having osteopenia or osteoporosis in men and women, respectively (men, 95% CI = 1.25–2.13, *p* = 0.004; women, 95% CI = 1.19–2.42, *p* = 0.004), which suggested that a higher level of ucOC was associated with the higher risk of osteopenia or osteoporosis in both men and women.

**Table 4:** Odds ratio of prevalent osteopenia or osteoporosis according to a 1 − SD increase in ucOC and BTMs in the men and women.

Variables	Men (n = 411)				Women (n = 468)			
	Unadjusted		Adjusted		Unadjusted		Adjusted	
	OR (95% CI)	*p*-Value	OR (95% CI)	*p*-Value	OR (95% CI)	*p*-Value	OR (95% CI)	*p*-Value
ucOC (ng/ml)	1.46 (1.19, 1.80)	**<0.001**	1.63 (1.25, 2.13)	**0.004**	1.07 (0.89, 1.29)	0.461	1.70 (1.19, 2.42)	**0.004**
P1NP (ng/ml)	1.27 (0.98, 1.65)	0.071	1.55 (1.07, 2.30)	**0.019**	3.91 (2.67, 5.73)	**<0.001**	2.47 (1.38, 4.45)	**0.003**
β-CTX (ng/ml)	0.68 (0.54, 0.87)	**0.002**	1.10 (0.87, 1.37)	0.462	3.77 (2.76, 5.14)	**<0.001**	3.13 (1.92, 5.10)	**<0.001**

For the unadjusted models, ucOC, P1NP, and β-CTX were analyzed separately; for adjusted model, age, BMI, Cr, and 25OHD were adjusted.

ucOC, undercarboxylated osteocalcin; P1NP, procollagen type 1 N-propeptide; β-CrossLaps of type I collagen-containing cross-linked C-telopeptide; BMI, body mass index; Cr, creatinine; 25OHD, 25-hydroxyvitamin D.

## Discussion

This was a large cross-sectional study exploring the profile of serum ucOC levels according to gender and age in the Chinese population. First, we found that men had a higher serum ucOC level than women after adjusting for age, BMI, Cr, and 25OHD, which was consistent with previous studies (28–30), but some studies also showed contrary results (31). The difference may partly be attributed to the difference in the hormonal milieu between genders. Foo et al. (28) believed that female sex hormones might have a role in modulating the level of ucOC, while Hiam et al. (30) found that testosterone may mediate the differences in ucOC levels between men and women. The above discrepancies require further study to elucidate the role of sex steroids in bone metabolism and explain these sex differences. Moreover, we found that in both men and women, ucOC levels decreased with age from youth, reaching the bottom values at the age of 40–49 years, and then increased in the subsequent life. The age-related changes in serum ucOC in the current study were consistent with the “U-shaped” relationship between ucOC and age in adult men proposed by Smith et al. (15). Perhaps due to different populations and sample sizes, the age with the lowest ucOC level in our study was 5 years earlier than that in Smith’s study. Similar age-related changes were also reported in other BTMs, like total OC, P1NP, and β-CTX, which may be related to different bone statuses during the life span (32). In youth, the higher ucOC levels might be correlated with beneficial increases in bone formation and suggested that the skeletal system had not yet reached maturity, while in the elderly, it might reflect the imbalance or uncoupling of bone resorption and formation and be correlated with bone loss (33).

Subsequently, we explored the associations of ucOC with BMD and BTMs in the Chinese population. First, we found that a higher ucOC level was correlated with lower BMD at the lumbar spine, femoral neck, and total hip in both men and women. Ito et al. (34) and Vergnaud et al. (20) found that ucOC was significantly and negatively correlated with femoral neck BMD, while Yamauchi et al. demonstrated that it was only negatively correlated with lumbar BMD (25). However, Liu et al. (24) considered that the increase of ucOC might be related to the changes in bone quality rather than mineral density, as they found no associations between ucOC and BMD. Our findings were similar to the results of Szulc et al. (23) and Emaus et al. (35) that elderly women with abnormally high serum ucOC have lower BMD values at all sites. Cummings et al. (36) found that a 26% decrease in femoral neck BMD of patients with abnormal levels of ucOC corresponds to a five- to sevenfold increase in hip fracture from the prospective data. Although the reason for the strong association between ucOC and BMD is not clear, the phenomenon might indicate that increased serum ucOC reflected intrinsic abnormalities of the bone matrix leading to bone loss and increased fragility. Additionally, we showed that a higher ucOC level was correlated with higher P1NP and β-CTX levels in both men and women, while Yamauchi et al. (25) demonstrated a strong and positive correlation between ucOC and uNTX in postmenopausal women. Although detailed mechanisms have not been clarified, one hypothesis may partly explain the positive correlation. When bone turnover is accelerated, markers including P1NP, β-CTX, and total OC are excessively synthesized, and greater quantities of vitamin K may be required. The relative insufficiency of vitamin K and increased OC are likely to cause increased ucOC levels (25). Since vitamin D is one of the main drivers of bone mineralization, we further evaluated the correlation of ucOC with BMD and BTMs in the vitamin D-sufficient subjects. In this study, we found that a higher ucOC level was still associated with a lower BMD and higher levels of P1NP and β-CTX.

Moreover, we found that a higher level of ucOC was associated with a higher risk of osteopenia or osteoporosis in both men and women, after adjusting for age, BMI, Cr, and 25OHD. Horiuchi et al. (26) revealed that serum ucOC level in individuals with osteopenia or osteoporosis was significantly higher than that in individuals with normal BMD, and Vergnaud et al. (20) considered that a higher ucOC level was a predictor of hip fracture in older women. However, currently, the relationship between ucOC levels and the risk of prevalent osteopenia or osteoporosis remained unclear. A hypothesis may partly explain the role of ucOC in the skeleton; that is, an abnormally high level of ucOC can reflect the accelerated bone turnover and cause bone loss and decreased BMD, which in turn leads to osteopenia or osteoporosis and serious fractures. Further randomized controlled studies or prospective studies are needed to reveal the specific causality and mechanism.

There were also some limitations in our study. First, some important variables were lacking, such as vitamin K, which acted as a coenzyme of carboxylase and catalyzed the carboxylation of OC. Our study did not evaluate the effect of vitamin K. Second, we used the commercial ELISA kit to measure the level of ucOC in serum, which was not considered the gold standard. Therefore, the serum ucOC level in this study could not reflect the levels measured by other technologies. Third, this is a cross-sectional study; thus, the proven causality between the ucOC, and BMD, BTMs, and prevalence of osteopenia and osteoporosis was limited. Therefore, further studies with a large sample size, wide age span, appropriate gender distribution, and unified measurement are still needed.

In conclusion, we first revealed the profile of the serum ucOC level according to gender and age in the Chinese population and demonstrated the associations of ucOC with BMD, BTMs, and the risk of prevalent osteopenia or osteoporosis. Our findings may provide a clue to elucidate the function of ucOC in bone metabolism.

## Data Availability Statement

The raw data supporting the conclusions of this article will be made available by the authors, without undue reservation.

## Ethics Statement

The studies involving human participants were reviewed and approved by the ethics committee of the Human Research of the Shanghai Jiao Tong University Affiliated Sixth People’s Hospital. The patients/participants provided their written informed consent to participate in this study.

## Author Contributions

Study design: YX and ZZ. Study conduct: YX, LL, and WH. Data analysis and interpretation: YX and LS. Manuscript draft: YX and LS. Manuscript content revision: WH, ZZ, and LL, YX and LS contributed equally to this work. All authors contributed to the article and approved the submitted version.

## Funding

This study was supported by the National Key R&D Program of China (2018YFA0800801), Shanghai Municipal Key Clinical Specialty, Key Specialty Construction Project of Pudong Health and Family Planning Commission of Shanghai (PWZzk2017-29), Outstanding Leaders Training Program of Pudong Health Bureau of Shanghai (PWRI2018-02), and Science Foundation of Shanghai Jiao Tong University Affiliated Sixth People’s Hospital (lygl202101).

## Conflict of Interest

The authors declare that the research was conducted in the absence of any commercial or financial relationships that could be construed as a potential conflict of interest.

## Publisher’s Note

All claims expressed in this article are solely those of the authors and do not necessarily represent those of their affiliated organizations, or those of the publisher, the editors and the reviewers. Any product that may be evaluated in this article, or claim that may be made by its manufacturer, is not guaranteed or endorsed by the publisher.

## Supplementary Material

Supplementary Figure 1 The profile of serum ucOC levels according to age in both men and women adjusted for BMI, Cr and 25OHD. Serum ucOC levels in the age group of 40-49 yrs. in both men were the lowest. The ucOC level decreased with increasing age until the age of 49 yr. and then quickly increased over the age of 50 yr.
