# Supplementary figures and images for: Undercarboxylated Osteocalcin and Its Associations With Bone Mineral Density, Bone Turnover Markers, and Prevalence of Osteopenia and Osteoporosis in Chinese Population: A Cross-Sectional Study
Source: Front Endocrinol (Lausanne). 2022 Jul 8;13:843912. doi: 10.3389/fendo.2022.843912 (PMC9309304; doi:10.3389/fendo.2022.843912)

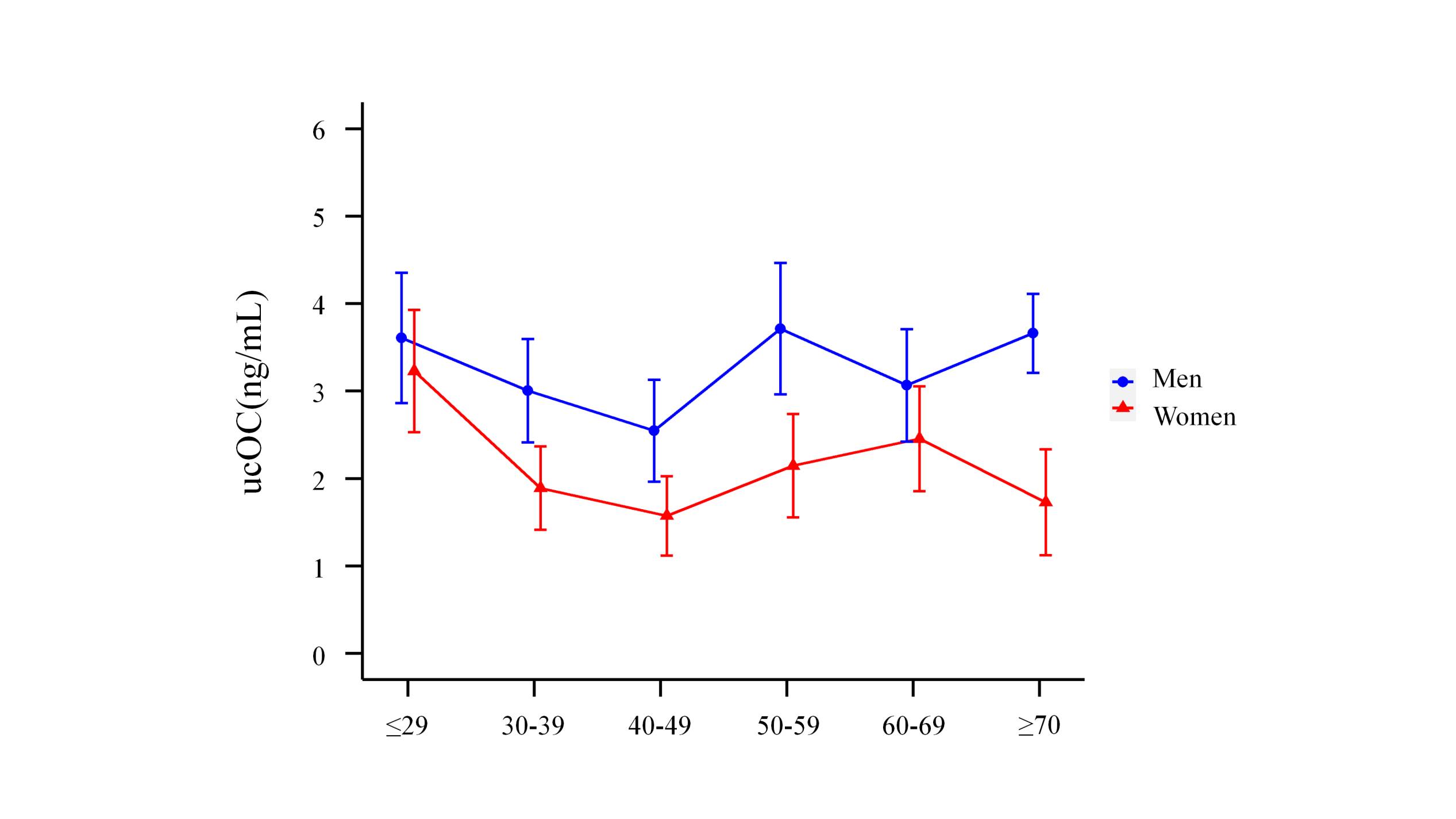

Supplement: Supplementary Figure 1 — The profile of serum ucOC levels according to age in both men and women adjusted for BMI, Cr and 25OHD. Serum ucOC levels in the age group of 40-49 yrs. in both men were the lowest. The ucOC level decreased with increasing age until the age of 49 yr. and then quickly increased over the age of 50 yr. [file Image_1.jpeg]
