# Supplementary material for: Undercarboxylated Osteocalcin and Its Associations With Bone Mineral Density, Bone Turnover Markers, and Prevalence of Osteopenia and Osteoporosis in Chinese Population: A Cross-Sectional Study
Source: Front Endocrinol (Lausanne). 2022 Jul 8;13:843912. doi: 10.3389/fendo.2022.843912 (PMC9309304; doi:10.3389/fendo.2022.843912)
Supplement: Supplementary file 2 [file Table_1.docx]

Supplemental Table S1. Correlations between serum ucOC level with BMD and BTMs in vitamin D-sufficient subjects

| Variables | Men(n=239) | | Women(n=273) | |
| --- | --- | --- | --- | --- |
|  | r | *p* | r | *p* |
| Lumbar spine BMD (g/cm^2^) | -0.145 | **0.035** | -0.273 | **<0.001** |
| Femoral neck BMD (g/cm^2^) | -0.078 | 0.251 | -0.150 | **0.016** |
| Total hip BMD (g/cm^2^) | -0.108 | 0.112 | -0.160 | **0.010** |
| P1NP (ng/mL) | 0.260 | **<0.001** | 0.383 | **<0.001** |
| β-CTX (ng/mL) | 0.156 | **0.022** | 0.403 | **<0.001** |

Abbreviations: BMD, bone mineral density; P1NP, procollagen type 1 N-propeptide; β-CTX, type I collagen containing cross-linked C-telopeptide. Adjusted for age, BMI, Cr, and 25OHD, and significant values (*p*<0.05) are presented in bold.
